# Ceruloplasmin is associated with the infiltration of immune cells and acts as a prognostic biomarker in patients suffering from glioma

**DOI:** 10.3389/fphar.2023.1249650

**Published:** 2023-08-11

**Authors:** Miaomiao Jia, Tianyu Dong, Yangyang Cheng, Fanghao Rong, Jiamin Zhang, Wei Lv, Shuman Zhen, Xianxian Jia, Bin Cong, Yuming Wu, Huixian Cui, Peipei Hao

**Affiliations:** ^1^ Department of Human Anatomy, Hebei Medical University, Shijiazhuang, Hebei, China; ^2^ International Cooperation Laboratory of Stem Cell Research, Shijiazhuang, China; ^3^ Postdoctoral Mobile Station of Biology, Hebei Medical University, Shijiazhuang, Hebei, China; ^4^ Research Unit of Digestive Tract Microecosystem Pharmacology and Toxicology, Chinese Academy of Medical Sciences, Hebei Medical University, Shijiazhuang, Hebei, China; ^5^ Institute of Medicinal Biotechnology, Chinese Academy of Medical Sciences and Peking Union Medical College, Beijing, China; ^6^ Fourth Hospital of Hebei Medical University, Shijiazhuang, Hebei, China; ^7^ Hebei Collaborative Innovation Center for Cardio Cerebrovascular Disease, Department of Physiology, Hebei Medical University, Shijiazhuang, China; ^8^ Hebei Key Laboratory of Neurodegenerative Disease Mechanism, Shijiazhuang, China

**Keywords:** gliomas, ceruloplasmin (CP), The Cancer Genome Atlas (TCGA), Chinese Glioma Genome Atlas (CGGA), tumor immune microenvironment (TIME)

## Abstract

Glioma is regarded as a prevalent form of cancer that affects the Central Nervous System (CNS), with an aggressive growth pattern and a low clinical cure rate. Despite the advancement of the treatment strategy of surgical resection, chemoradiotherapy and immunotherapy in the last decade, the clinical outcome is still grim, which is ascribed to the low immunogenicity and tumor microenvironment (TME) of glioma. The multifunctional molecule, called ceruloplasmin (CP) is involved in iron metabolism. Its expression pattern, prognostic significance, and association with the immune cells in gliomas have not been thoroughly investigated. Studies using a variety of databases, including Chinese Glioma Genome Atlas (CGGA), The Cancer Genome Atlas (TCGA), and Gliovis, showed that the mRNA and protein expression levels of CP in patients suffering from glioma increased significantly with an increasing glioma grade. Kaplan-Meier (KM) curves and statistical tests highlighted a significant reduction in survival time of patients with elevated CP expression levels. According to Cox regression analysis, CP can be utilized as a stand-alone predictive biomarker in patients suffering from glioma. A significant association between CP expression and numerous immune-related pathways was found after analyzing the data using the Gene Ontology (GO), Kyoto Encyclopedia of Genes and Genomes (KEGG), and Gene Set Enrichment Analysis (GSEA). Tumor Immune Estimation Resource (TIMER) and CIBERSORT analyses indicated a substantial correlation between the CP expression and infiltration of immunocytes in the TME. Additionally, immune checkpoints and CP expression in gliomas showed a favorable correlation. According to these results, patients with glioma have better prognoses and levels of tumor immune cell infiltration when their CP expression is low. As a result, CP could be used as a probable therapeutic target for gliomas and potentially anticipate the effectiveness of immunotherapy.

## 1 Introduction

Glioma is the most commonly occurring and malignant adult brain tumor (Xu et al., 2023), and divided into ependymoma, astrocytoma [including glioblastomas (GBM)], oligodendroglioma, mixed glioma, and a few other tumors that vary widely in histology (from benign ependymoma to the most aggressive and lethal grade-IV GBM) (Antin et al., 2020; Walsh et al., 2023). Despite multiple conventional therapy, including surgical resection, chemoradiotherapy and immunotherapy, glioma still possess a high recurrence and fatality rate (Dong et al., 2023). The impact of immunotherapy on glioma has become one of the hottest areas of research owing to its effectiveness in treating various solid tumors and hematological cancers in recent years (Chang et al., 2023; Hovhannisyan et al., 2023). The advancement of genomics has led to the identification of some molecular markers associated with specific gliomas phenotypes such as isocitrate dehydrogenase (IDH) (Nuechterlein et al., 2021), phosphoinositide 3-kinase (PI3K) (Yan et al., 2020), telomerase reverse transcriptase (TERT) (Park et al., 2022), and phosphatase and tensin homolog (PTEN) (Cheng et al., 2021). However, there is a shortage of reliable biomarkers that can effectively predict the prognosis of glioma patients. Therefore, the search for new immune-related molecular biomarkers affecting glioma is of great importance for its treatment.

Iron, an essential component of hemoglobin, plays a key role in the cell proliferation and differentiation pathways and participates in numerous important physiological processes such as electron transfer, cellular respiration, energy metabolism, and detoxification by catalyzing redox reactions (Meheust et al., 2021; Dugan et al., 2022). Dysregulated iron metabolism can cause accumulation of intracellular ferrous iron (Fe^2+^), and the overloaded free Fe^2+^ in the cell can generate a large amount of ROS such as hydroxyl radicals through Fenton reaction (Huang et al., 2023a). Excessive ROS can cause oxidative stress, adversely affect the stability of the genome, possibly induce malignant transformation, and also trigger apoptosis signal cascade reaction and ferroptosis. In addition, some studies have proved that the innate immune cells resident in the tumor microenvironment, such as macrophages and neutrophils, are the source of iron and iron related proteins, and can activate the signal pathway controlling iron metabolism and promoting the imbalance of iron metabolism in tumor cells (Weston et al., 2016; Su et al., 2023). Therefore, changes in iron metabolism are one of the important characteristics of tumors and are related to their occurrence and development (Wang et al., 2023). Jaksch-Bogensperger et al. observed higher serum ferritin levels in patients with high-grade glioma (Jaksch-Bogensperger et al., 2020). By increasing the expression of their transferrin Receptor 1 and ferritin genes, GBM stem-like cells are better able to absorb iron from the microenvironment (Pandya Shesh et al., 2023). Additionally, in high-grade gliomas, hypoxia-induced expression of the ferritin light chains is also related to the epithelial-mesenchymal transition (EMT) and chemoresistance (Liu et al., 2020). Current research and drug discovery in iron metabolism have provided effective therapeutic target and a potential prognostic biomarker for glioma (Tan et al., 2023; Zhang et al., 2023).

Ceruloplasmin (CP), an abundant serum α2-glycoprotein and a key iron oxidase in the human body can catalyze divalent iron to trivalent iron, thus promoting iron binding to transferrin (Pal et al., 2022). Since only trivalent iron can bind to transferrin, and CP is essential in the formation of trivalent iron, CP plays a crucial role in iron transport and iron homeostasis in the body (Thepsuwan et al., 2023). Studies have shown that CP in the brain promotes both iron release and iron uptake in brain cells (Squitti et al., 2023). Besides, CP plays a more prominent role in iron uptake than release (Mukhopadhyay et al., 2000; Mietto et al., 2021). Some recent studies stated that the abnormal CP expression occurring in the brain could be attributed to a few neurological diseases (Klomp and Gitlin, 1996; Qian and Ke, 2019; Villadsen et al., 2023). At present, the existing literature studies have reported that in the iron-deficient and iron-saturated cells, CP, regardless of concentration, promotes the uptake of transferrin-bound iron and ferric citrate by glioma cells (Attieh et al., 1999; Roy et al., 2022). Moreover, high expression of CP in tumor cells, such as lung cancer (Chang et al., 2022), liver cancer (Shang et al., 2020), melanoma (Liu et al., 2022a), and breast cancer (Chen et al., 2021), can inhibit ferroptosis, thus reduces tumor cell death and promotes tumor progression. High expression CP in tumor cells transfers electrons to oxygen and oxidizes Fe^2+^ to Fe^3+^ via regulating copper, thus reducing intracellular Fe^2+^ and inhibiting ferroptosis (Li et al., 2020). CP, which is a potential oncogenic factor, is aberrantly expressed in various malignancies like lung cancer (Tsai et al., 2020), ovarian cancer (Dai et al., 2020), and hepatic cancer (Shang et al., 2020), and can be involved in tumor growth and metastasis by regulating ferroptosis, angiogenesis, and tumor microenvironment (TME).

CP expression and carcinogenesis are known to be closely related, although there is currently no information on how CP contributes to the pathophysiology, clinical relevance of glioma or its prognosis. This research used a variety of bioinformatics software and tools to analyze the relationship between CP expression with the clinical feature, patient survival and infiltration of immune cells. Besides, the role of CP in glioma behavior was also presented. CP expression was upregulated significantly in glioma tissues in comparison to non-tumor tissues and increased with glioma grades. The prognosis of the patients suffering from glioma was inversely linked to the high CP expression. Additionally, there was a direct correlation between CP expression and antitumor immune responses, such as immunosuppressive targets and the infiltration of immune cells (including neutrophils, macrophages, dendritic cells, CD4^+^ T cells, CD8^+^ T cells and B cells). These findings emphasize the critical function of CP in carcinogenesis and suggest that CP may be essential in the immune landscapes of gliomas TME.

## 2 Materials and methods

### 2.1 Raw data acquisition

Two separate databases, the Chinese Glioma Genome Atlas (CGGA) (dataset 1, *n* = 325) and the CGGA (dataset 2, *n* = 693), provided the RNA sequencing information for patients with diffuse glioma (Zhao et al., 2021). The Cancer Genome Atlas (TCGA) was used to retrieve the glioma patient dataset (*n* = 698), which included the clinical data and gene expression profiles (Blum et al., 2018). Then, from TCGA, CP expression data (normalized into Fragments Per Kilobase per Million [FPKM]) and all clinical information were also retrieved. The clinicopathological data that was acquired, including grade, age, sex, survival status, and overall survival (OS), were combined. Finally, default value samples were removed according to the statistical needs of the follow-up study.

### 2.2 Relationship between CP expression and glioma grade

Expression data of CP in 31 cancers were obtained from TCGA, analyzed by the HiPlot open-source web platform UCSCXenaShiny module (https://hiplot.com.cn/advance/ucscxena), and grouped into paraneoplastic tissues and tumor tissues. Expression and clinical characteristics of CP genes in TCGA, CGGA, Gravendeel, and Rembrandt glioma databases were obtained through the Gliovis web platform (http://gliovis. Bioinfo.cnio.es/). In addition, the association between the glioma grade, CP expression, and patient prognosis were analyzed. Finally, the CP expression in the normal, low-grade, and high-grade glioma tissues were studied based on the HPA website.

### 2.3 Correlation between the CP expression and OS of the patients with glioma

FIn order to investigate the correlation between the CP expression and OS of patients with glioma, samples obtained from different glioma-related databases were categorized into the high-CP and low-CP expression categories, according to the median of CP expression. The Kaplan-Meier (KM) survival curves were plotted based on the survival data of the Gliovis patients, based on the information derived from the TCGA, CGGA, Gravendeel, and Rembrandt databases, followed by log-rank and Wilcoxon tests. Events in the high-CP and low-CP expression categories in every dataset were pooled. Subsequently, the relative survival risk ratio (RR) of every group in these databases were calculated by using the binary data meta-analysis tool on the HiPlot web platform. Finally, forest plots of risk factors affecting patient survival were drawn for every database.

### 2.4 Association between the CP expression and clinical feature

First, the association between the CP expression in glioma and clinical features such as sex, age, glioma subtype, IDH mutation and 1P19q deletion status, was investigated. The CP expression heat map was mapped by the R package “ComplexHeatmap”. Afterward, the variations in the clinical feature between the high-CP and low-CP expression groups were investigated, based on the CGGA and TCGA datasets, and subjected to COX regression-based independent prognostic analysis to observe the correlation between the CP expression and the prognosis of glioma patients. Finally, a novel nomogram model incorporating clinical information and the CP expression data was developed, using the R language. Overall, the nomogram model performed better than the conventional staging system in predicting prognosis.

### 2.5 Gene-gene and protein-protein interaction networks of CP

The GeneMANIA cohort (http://www.genemania.org) was utilized to construct the CP interaction network. In addition, a Protein-Protein Interaction (PPI) network for CP was designed with the help of the STRING online database (https://string-db.org/). The correlation between the CP and iron metabolism-linked genes in glioma was studied using the TCGA, low-grade glioma (LGG), and GBM databases, based on the Timer (https://cistrome.shinyapps.io/timer/) network platform.

### 2.6 Functional analysis of the differentially expressed genes in the High-CP and Low-CP expression groups in the CGGA and TCGA datasets

Relevant genes co-expressed with CP in the CGGA database were identified by co-expression analysis. Representative genes significantly associated with CP were visualized in circles using the R package “corplot” and the package “Circlize”. The samples from CGGA and TCGA were classified into 2 groups based on the CP expression. The LIMMA program package, using the R language, was employed for screening the Differentially Expressed Genes (DEGs) in the two groups, and the heat map was drawn with the pheatmap program package. KEGG and GO enrichment analyses of the DEGs in CGGA and TCGA databases were performed using R language to elucidate the biological functions and pathways involving CP. Subsequently, GSEA enrichment analysis of CP was performed using the c5.go.v7.4.symbols and c2.cp.kegg.v7.4symbents datasets. The groups with the Nominal (NOM) *p*-values and the False Discovery Rate (FDR) q-values ≤0.05 were defined as significantly enriched groups.

### 2.7 Correlation between the CP and antitumor immunity

Depending on the median level of CP expression, the samples were sorted into the high-CP and low-CP expression groups. R language tools were used to examine the Tumor Mutation Burden (TMB) and the TME scores of both groups. In addition, the Timer web platform (https://cistrome.shinyapps.io/timer/) was used to thoroughly assess the landscape of immune cell infiltration. Using the TCGA database, the “Gene” module of the web platform was utilized to examine the correlation between the CP expression and the immune cell infiltration (including macrophages, dendritic cells, neutrophils, CD4^+^ T cells, CD8^+^ T cells and B cells). Immune checkpoint medications that target PD-1, PD-L1, and CTLA-4 are currently showing proving to be effective while treating cancer patients. The GEPIA web platform was used to ascertain the association between CP and PD-L1, PD-1, and CTLA-4 in the GBM and LGG of TCGA. In addition, the GBM dataset derived from the TCIA web portal (https://tcia.at/home) was used to examine the effectiveness of anti-PD-1 and CTLA-4 therapy in the patients categorized into the two groups.

### 2.8 Relative abundance of the infiltrating immune cells in the TME

The CiberSort web platform, which uses a deconvolution algorithm based on the gene expression that was based on the gene annotation matrix of 22 types of immune cell, was used to examine the relationship between the CP expression and the Tumor-Infiltrating Immune Cells (TIICs) in glioma. *p*-values were computed for each sample in the CGGA dataset. Additionally, the R software was used to display the association between the immune cells in every dataset. The composition of the infiltrating immune cells of each sample can be identified using CiberSort. As a result, the relative quantities of various immune cells in every group can be compared effectively, and the outcomes were plotted as a box plot. The association between immune cells and CP expression was then used to construct a lollipop plot showing the correlation between the immune cells.

### 2.9 Statistical analysis

Data were presented as the mean ± standard deviation (SD). The Student’s *t*-test was used to evaluate whether there were any significant variations between the two groups. To compare more than two groups, a one-way Analysis of Variance (ANOVA) was used. Correlation analysis was conducted using Spearman’s technique. In accordance with the 50% cut-off for gene expression, patients were sorted into high-CP and low-CP expression subtypes. The KM survival analysis and the log-rank significance tests were employed to compare the OS rates between the 3 groups. Additionally, COX regression models were utilized to examine the prognosis-influencing components. A software called GraphPad Prism 9.0 was used to create the graphs (GraphPad Inc., San Diego, United States).

## 3 Results

### 3.1 Elevated CP expression level with the increase of glioma grade

**FIGURE 1:**
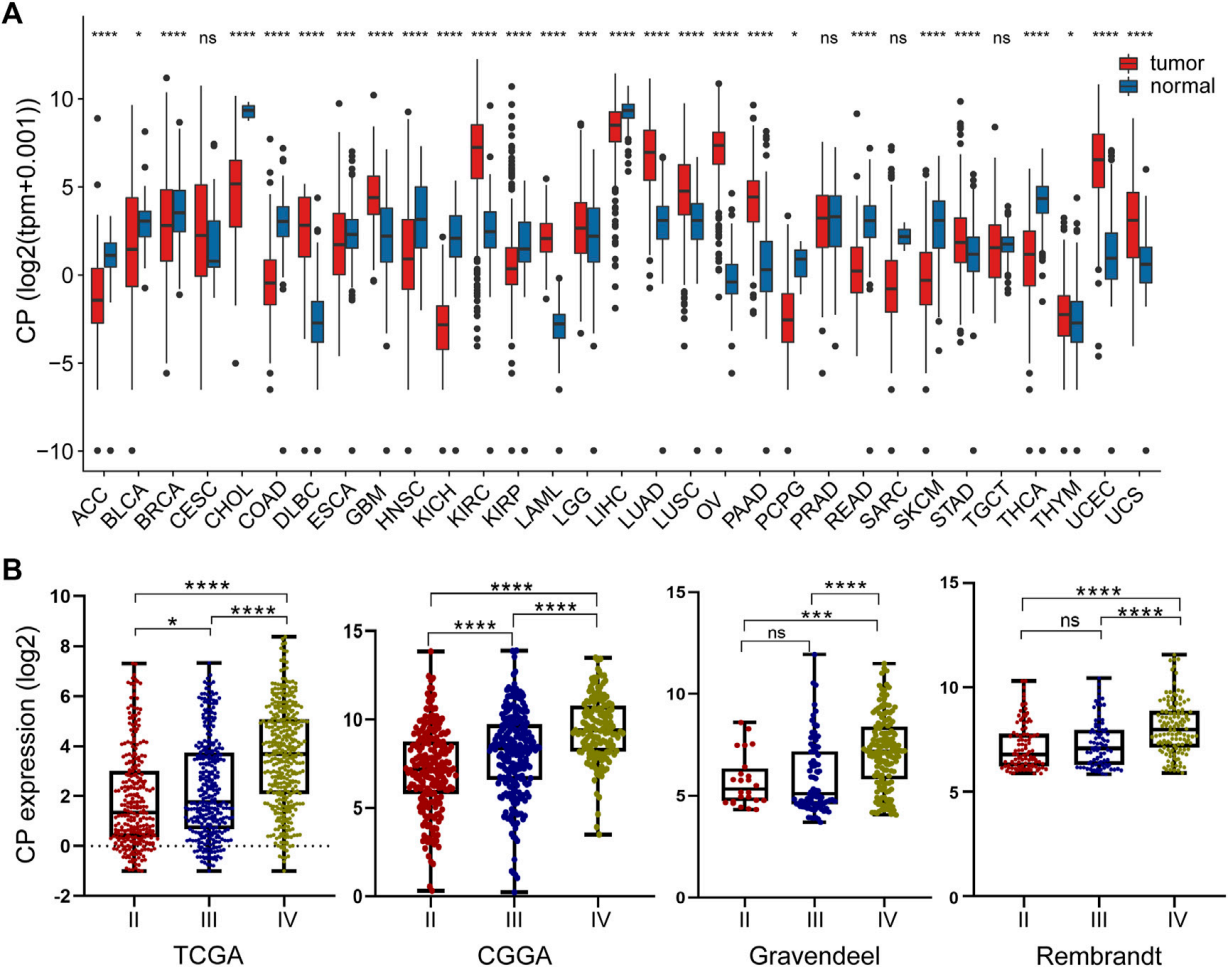
Analysis of the relationship between CP expression and glioma grade. **(A)** Pan-cancer analysis of CP expression in the TCGA and GTEx databases. **(B)** Relationship between the expression of CP and grade of glioma. (Note: ***p < 0.001 and *p < 0.05.).

Pan-cancer analysis suggested significant differences in CP expression between a variety of tumor tissues and paraneoplastic tissues. CP expression was higher in GBM, LGG, ovarian cancer (OV), kidney renal clear cell carcinoma (KIRC), and other tumor tissue samples compared to paraneoplastic tissues (Figure 1A). The link between the CP expression and World Health Organization (WHO) grade in glioma was assessed using the TCGA, CGGA, Gravendeel, and Rembrandt glioma databases. The results revealed a significant increase in CP expression with relatively advanced glioma grade (Figure 1B).

### 3.2 Relationship between CP expression and clinical feature of patients suffering from glioma

**FIGURE 2:**
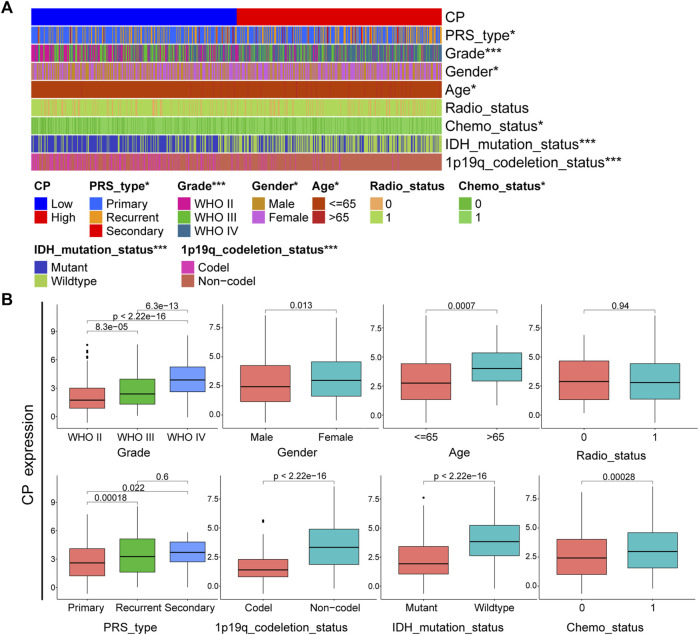
Analysis of the relationship between CP and clinical features in CGGA glioma dataset. **(A)** The relationship between CP expression and clinical features of CGGA dataset. **(B)** Each clinical feature was analyzed for differences in the CP high/low expression groups. (Note: ***p < 0.001 and *p < 0.05.).

According to the CGGA dataset, CP exhibits significant differences in PRS type, tumor grade, gender, age, chemotherapy status, IDH mutations and 1p19q deletion status (*p* < 0.001) (Figures 2A, B). Consistently, the CP expression was seen to be significantly differences (*p* < 0.001) in the glioma grade, gender and age, validated in the TCGA cohort (Supplementary Figures S1A, B). There is a lower expression of CP in low-grade, IDH-mutant, and chemotherapeutic sensitivity glioma. Elderly (aged over 65 years) and male patients was significantly higher. Besides, no matter male or female, those with high CP expression are more likely to be recurrent and secondary. CP might represent a new biomarker of prognosis and classification-based treatment in patients with gliomas.

### 3.3 Relationship between CP expression and glioma patient survival

**FIGURE 3:**
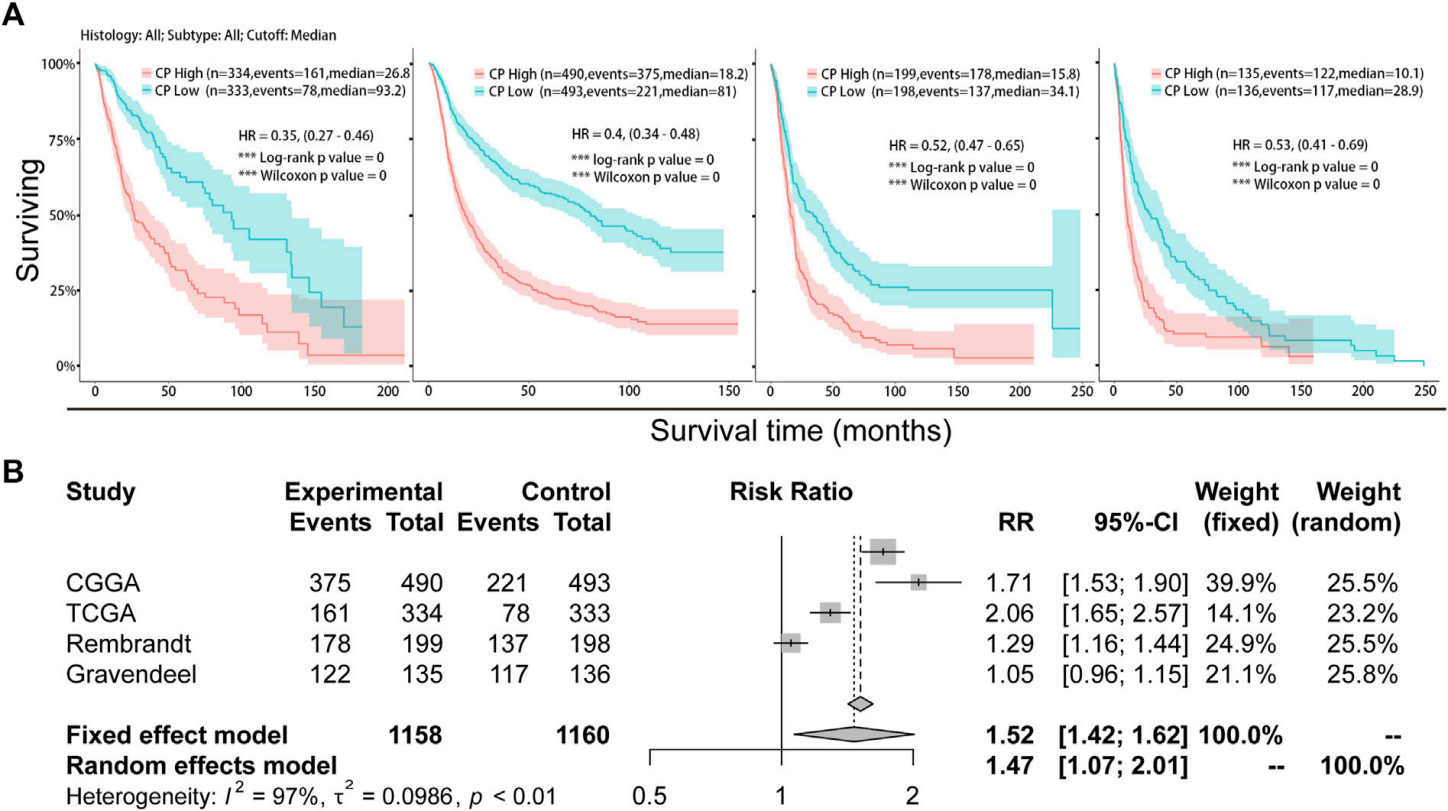
Analysis of the relationship between CP expression and survival in glioma. **(A)** Kaplan-Meier plots of CP in a variety of glioma datasets. The 95% confidence interval (CI) is shown. The patients were divided into high and low expression groups by the median expression level. **(B)** Forest plot of the risk ratio for patients with high CP expression levels compared to patients with low CP expression levels.

**FIGURE 4:**
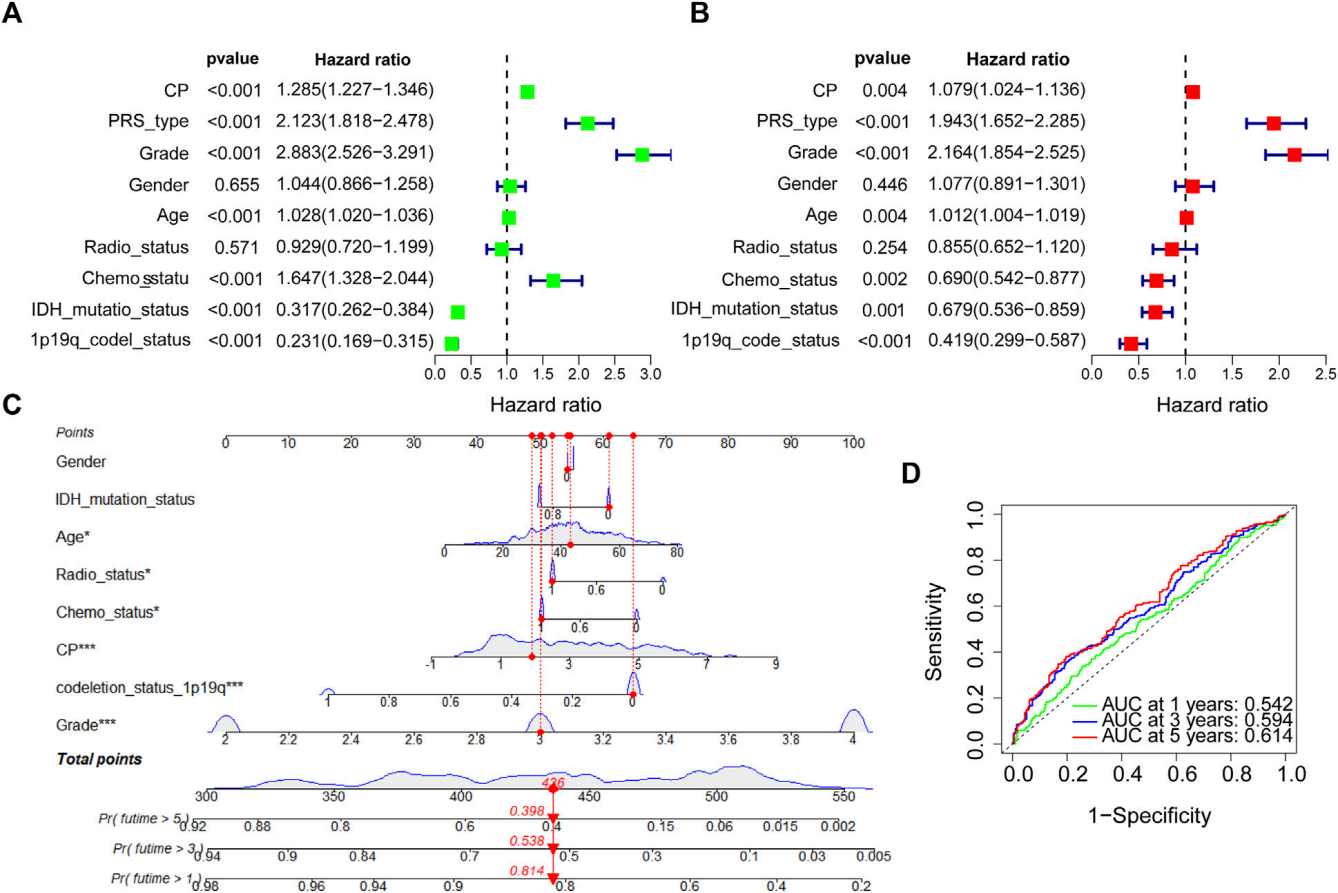
COX regression analysis and establishment of prognostic model in CGGA dataset. **(A)** Univariate analysis of CP in the CGGA dataset. **(B)** Multivariate analysis of CP in the CGGA dataset. **(C)** The nomogram was constructed based on four factors for predicting 1, 3 or 5 years survival in CGGA glioma patients. **(D)** The calibration plots of internal validation in CGGA showed well consistency in predicting 1, 3 or 5 years survival. (Note: ***p < 0.001 and *p < 0.05).

Samples from CGGA, TCGA, Rembrandt, and Gravendeel were sorted into the Low-CP and High-CP expression groups, depending on the median CP expression levels. KM curves and statistical tests revealed that the OS rates were reduced significantly in the High-CP expression category compared to the Low-CP expression category in all 4 datasets (*p* < 0.001) (Figure 3A). Considering the significant difference in sample size among the four datasets, RRs (Risk Rates) were calculated for Meta-analysis of the four datasets using a fixed-effects model to improve the reliability of the results. The Low-CP expression patients showed a significantly long OS duration compared to the High-CP expression patients (fixed-effects model: RR: 1.52; 95% Confidence Interval [CI]: 1.42/1.62) (Figure 3B). In the TCGA and CGGA databases, COX regression analysis revealed that CP was an independent factor that affected the prognosis of the patients suffering from glioma (*p* < 0.001) (Figures 4A, B and Supplementary Figures S2A, B). Overall, the nomogram model constructed based on CP expression achieved better prediction of prognosis (Figures 4C, D and Supplementary Figures S2A, B).

### 3.4 Gene-gene and PPI networks of CP

**FIGURE 5:**
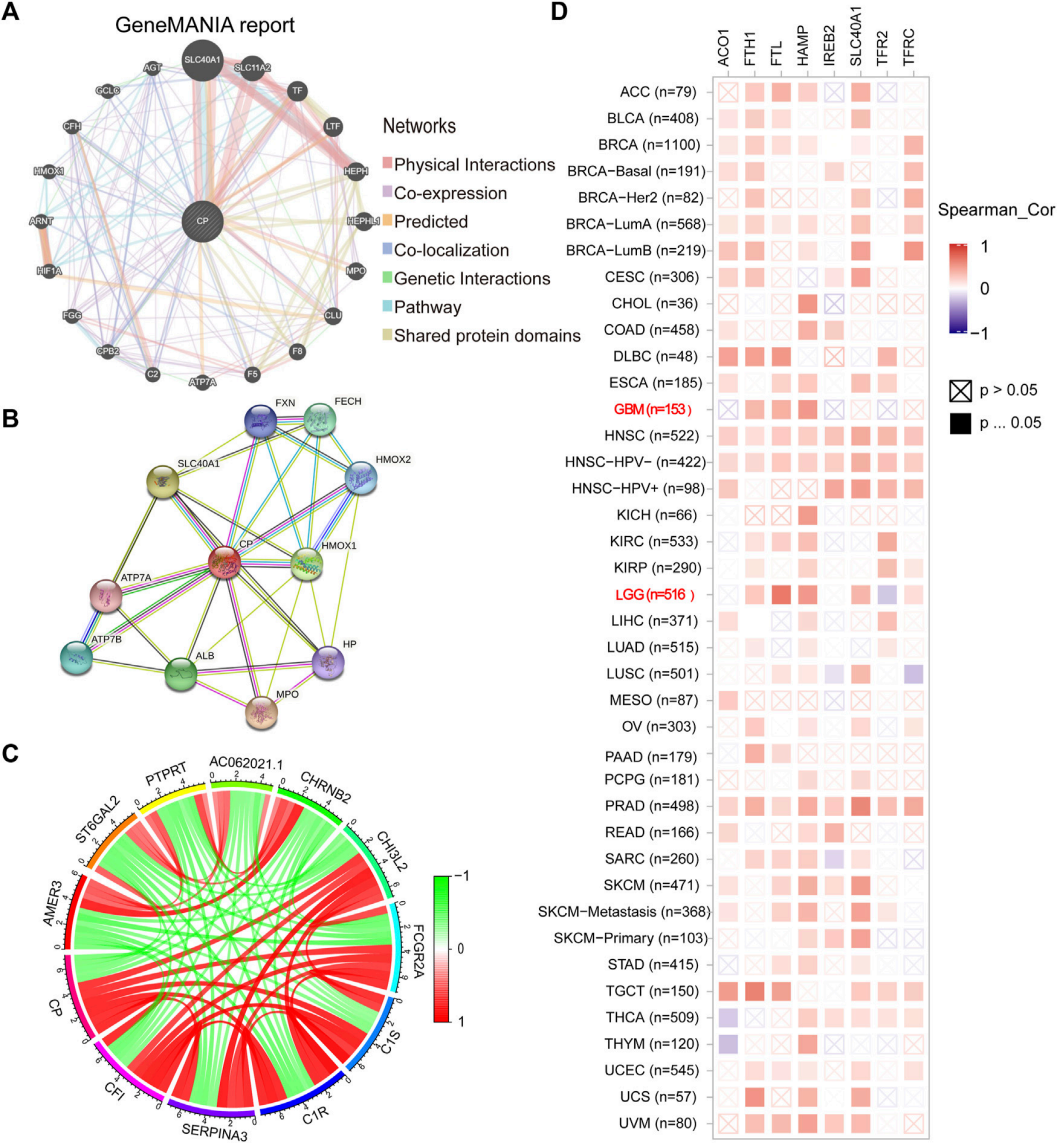
Analysis of CP-interacting genes and proteins. **(A)** The gene-gene interaction network of CP was constructed using GeneMANIA. **(B)** The PPI network of CP was generated using STRING. **(C)** The gene chord diagram of CP from co-expression analysis. Red lines represent positive correlations with CP, and green lines represent negative correlations with CP. **(D)** A heatmap shows the correlations between CP and iron metabolism-related genes in LGG and GBM.

The CP gene-gene interaction network was developed using GeneMania. Iron metabolism-related genes such as SLC40A1 were closely related to CP (Figure 5A). The PPI network for CP was designed using the STRING database. The most relevant genes primarily included iron metabolism-related genes such as SLC40A1, haptoglobin (Hp), and frataxin (FXN) (Figure 5B). The chord diagram displays the top 11 genes that were co-expressed with CP in the CGGA database (Figure 5C). CP had a positive correlation with CH3L2, FCGR2A, C1S, C1R, SERPINA3, and CF1, and a negative correlation with AMER3, ST6GAL2, PTPRT, AC062021.1, and CHRNB2 (Figure 5C). Furthermore, the correlation between CP expression and the iron metabolism-linked genes was investigated by TCGA database. CP showed a significantly positive link to the HAMP, FTH1, FTL, SLC40A1, TFRC, a negative correlation with RFR2 of LGG, and a significantly positive relationship with the HAMP, FTH1, and FTL of GBM (Figure 5D).

### 3.5 Functional evaluation of the DEGs in the High-CP and Low-CP expression categories

**FIGURE 6:**
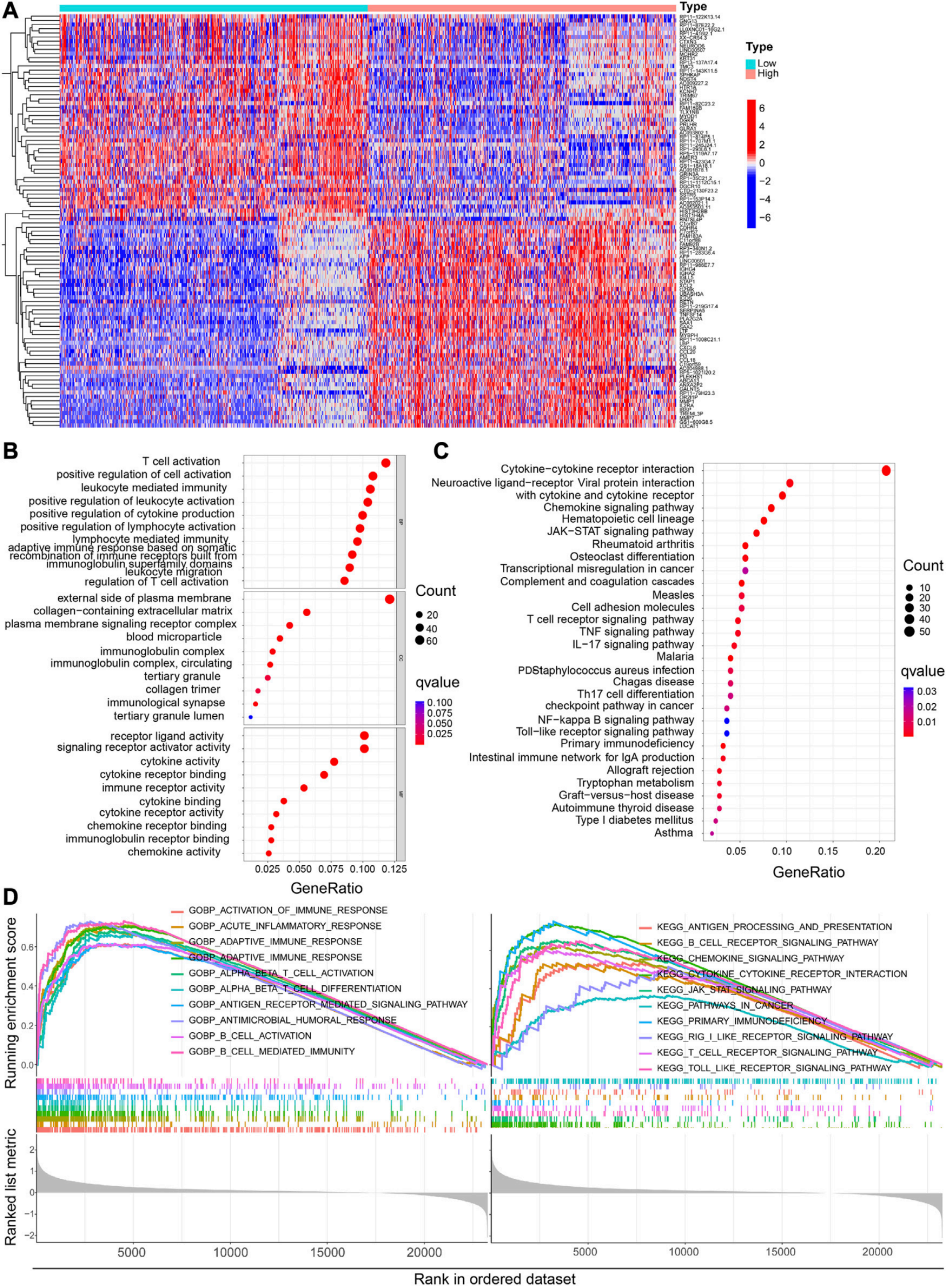
Functional analysis of DEGs between the high and low CP expression groups in the CGGA dataset. **(A)** Heatmaps of the differentially expressed genes between the high and low CP expression groups. GO **(B)** and KEGG **(C)** analyses of DEGs. **(D)** GSEA GO and KEGG enrichment analyses of the high and low CP expression groups in the CGGA dataset.

The heat map shows the Differentially Expressed Genes (DEGs) in the High-CP and Low-CP expression categories in the CGGA dataset (Figure 6A) and the TCGA dataset (Supplementary Figure S3A). The relevant pathways and biological functions of CP were investigated by KEGG and GO enrichment analyses, and the top 10 Biological Processes (BP), Cellular Components (CC), and Molecular Functions (MF) terms were listed. In the CGGA dataset, CP was seen to be enriched in the immune response-linked BP pathways, like T cell activation, lymphocyte mediated immunity, and the regulation of the T cell activation (Figure 6B). In addition, the top 10 KEGG pathways related to DEGs are presented in Figure 6C. Out of these pathways, many immune-linked pathways were related to CP, including PD-L1 expression, PD-1 checkpoint and T cell receptor signaling pathway in cancer (Figure 6C). Furthermore, GSEA-GO and KEGG analyses of the CGGA dataset implied that CP was associated with the primary immunodeficiency and adaptive immune-associated pathways, such as T cell and B cell activation, antigen receptor-mediated signaling pathway, T cell receptor signaling pathways (Figure 6D).

CP was primarily enriched in the immune response-linked BP pathways in the TCGA glioma database, including positive regulation of leukocyte activation, B cell-mediated immunity, and adaptive immune response (Supplementary Figure S3B). In addition, Supplementary Figure S3C displays important KEGG pathways associated with DEGs. Among these, many immune-related pathways, including the B cell/lymphocyte-mediated immunity and the B cell receptor signaling pathway, were significantly linked to CP. Additionally, GSEA-GO and KEGG analyses in the TCGA dataset revealed that CP was associated with the immune-linked or T cell- and B cell-linked pathways, such as B cell activation, B cell and T cell receptor signaling pathway (Supplementary Figure S3D).

### 3.6 Relationship between CP expression and immune microenvironment

**FIGURE 7:**
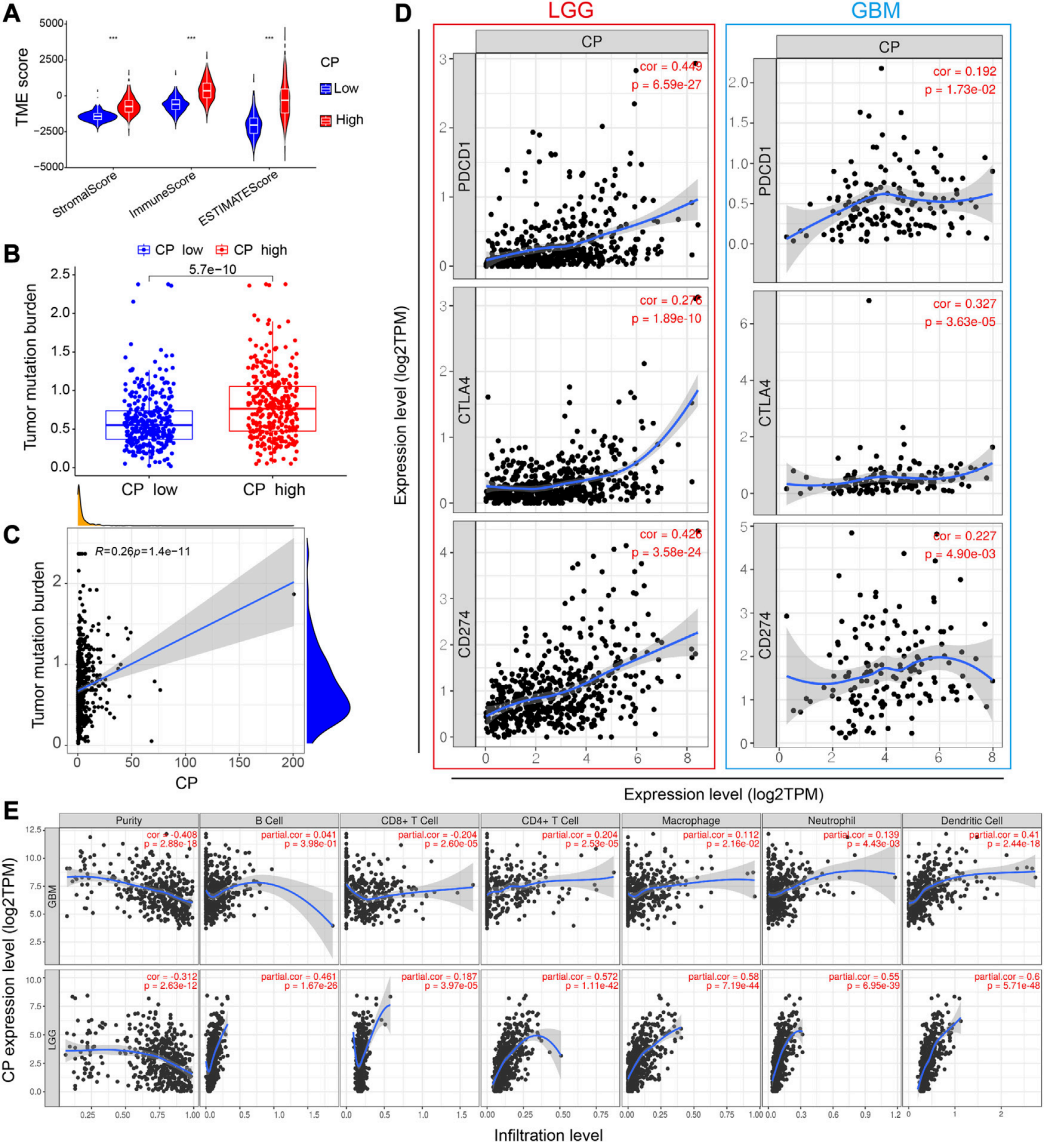
Analysis of the correlation between CP and immunity. **(A)** The TME scores between the high and low CP expression groups in the CGGA database. **(B)** The TMB between the high and low CP expression groups in the CGGA database. **(C)** The correlation between CP expression and TMB. **(D)** Scatterplots of the correlations between CP expression and PD-1, PD-L1 and CTLA-4 in LGG and GBM. **(E)** CP is significantly associated with tumor purity and is positively correlated with the infiltration of different immune cells according to the TIMER database.

**FIGURE 8:**
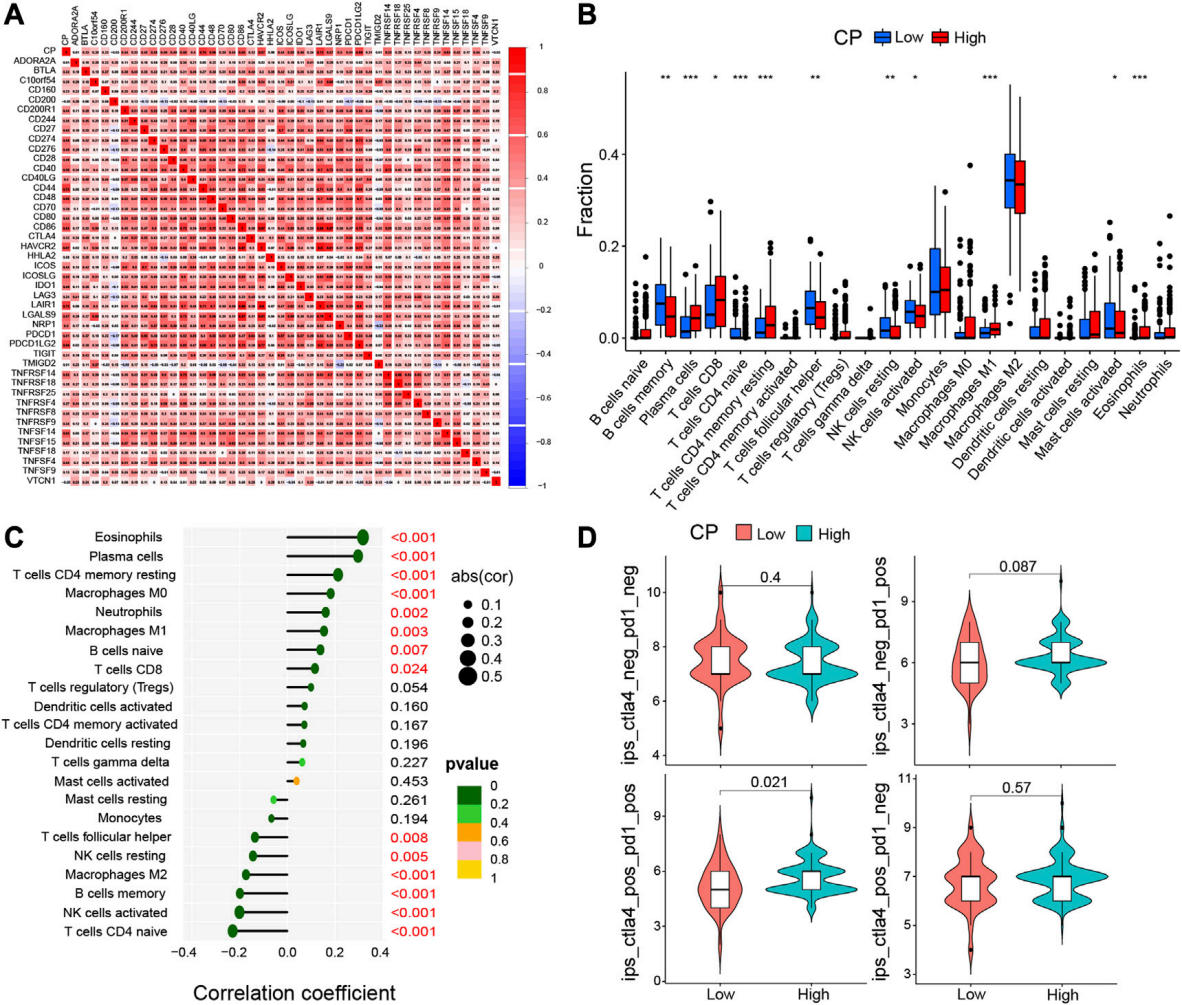
Immune cell infiltration analysis were calculated by the CIBERSORT algorithm and TIMER database. **(A)** In CGGA dataset, the correlation of each immune cell was analyzed. **(B)** The varied proportions of 22 subtypes of immune cells in the high and low CP groups in tumor samples. Horizontal and vertical axes represent TIICs and relative percentages, respectively. Blue and red colors represent the low and high CP expression groups, respectively. **(C)** The correlation between immune infiltrating cells and CP expression. The ordinate represents the name of the immune cell, and the abscissa represents the correlation coefficient. **(D)** In the GBM data set of the TCIA network platform (https://tcia.at/home), the efficacy of the high and low expression of CP in receiving the anti-PD-1 and anti-CTLA-4.

The immune and mesenchymal scores were calculated for each glioma sample in CGGA using the “Limma” and “Estimate” software packages in the R language to assess the proportion of immune and mesenchymal components in TME. The violin plot showed that the scores of TME were significantly elevated in the High-CP expression category compared to the Low-CP expression category (Figure 7A). The TMB scores were seen to be significantly increased in the High-CP expression category compared to the Low-CP expression category in the CGGA database, and the CP expression was positively correlated with TMB (Figures 7B, C). The correlation of well-known T cell checkpoints in the GEPIA database, such as PD-L1, PD-1, and CTLA-4 with the CP expression was further investigated. The expression of CP in LGG and GBM was significantly linked to the PD-L1, PD-1, and CTLA-4 expression (Figure 7D). In addition, the correlation of CP expression in LGG and GBM with 6 immune cells, including macrophages, neutrophils, dendritic cells, CD4^+^ T cells, and B cells, was analyzed by the Timer network platform. CP expression were significantly and positively correlated to the infiltration of CD4^+^ T cells, B cells, neutrophils, macrophages, and dendritic cells in GBM and negatively correlated to the CD8^+^ T cells (Figure 7E). Based on the CiberSort resource, the abundance ratios and relationship between each other of 22 types of immune cells in CGGA glioma samples were calculated (Figure 8A). The difference in the concentrations of 22 types of immune cells between the High-CP and Low-CP expression categories suggested that eosinophils, plasma cells, macrophages M0, T-cell CD4 memory resting, and neutrophils were positively and significantly related to the CP expression, whereas macrophages M2, NK-activated cells, B-memory cells, NK-resting cells, and other immune cells showed a significant and negative relation with CP expression (Figures 8B, C). In the GBM dataset of the TCIA web platform (https://tcia.at/home), patients in the high-CP expression group exhibited significant immune escape in CTLA-4 and PD-1 positive GBM tissues compared to the low-CP expression group after anti-PD-1 and CTLA-4 treatments (Figure 8D).

## 4 Discussion

Glioma is a serious disease that affects human health (Brown, 2023). For decades, a large number of studies on molecular markers and molecular targeted drugs only had a little effect in prolonging life expectancy of patients with glioma (Huang et al., 2023b). Therefore, searching for glioma-related molecular helps understand the mechanism of glioma occurrence and progression and provides molecular targeted therapeutic targets for glioma.

CP is an acute phase protein that is activated under a variety of circumstances, including inflammation, infection, diabetes, and trauma (Liu et al., 2022b). In the past few years, numerous physiological functions and activities of CP have been found, such as antioxidant activities, iron oxidase, copper transport, iron homeostasis management, and oxidation of organic amines (Hellman and Gitlin, 2002). Recent data implied that CP is linked to the onset and advancement of tumors. Lung (Dai et al., 2020; Schneider et al., 2022), epithelial ovarian (Schneider et al., 2022), colon (Lin et al., 2019), and bile duct tumors (Han et al., 2017) also have high serum CP levels. CP established the connection between the prognosis and severity of the above cancers via several molecular processes and signaling pathways. Intriguingly, in one study, the hypothesis suggested that it was associated with the local Fe homeostasis (Li et al., 2020). CP-ferroportin system (CP-Fpn) is the primary intracellular iron export pathway (Helman et al., 2023). Iron oxidation is another important function of CP. CP can transfer electrons to oxygen via regulating copper, which oxidizes Fe^2+^ to Fe^3+^ and enters the cell after binding to transferrin, thus providing sufficient iron for cell proliferation (Forciniti et al., 2020; Roy et al., 2022). Depletion of CP caused the accumulation of ferrous iron (Fe^2+^) in cytoplasm. The accumulated Fe^2+^ mediates the formation of reactive oxygen species *in vivo* by Fenton reaction, eventually leading to ferroptosis in cells (Huang et al., 2023a). In addition, CP expression is increased in tumor cells of different tumors like liver cancer, lung cancer, and melanoma, thus transferring electrons to oxygen and oxidizing Fe^2+^ to Fe^3+^ via regulating copper, thereby reducing intracellular Fe^2+^ and inhibiting ferroptosis (Li et al., 2020). Therefore, high CP expression in tumor cells inhibits ferroptosis, which reduces tumor cell death and promotes tumor progression. Although the role of CP in glioma is not clearly, CP is regarded as an attractive potential target for cancer treatment, and CP-inhibiting drugs are being investigated currently.

Here, we found that the CP expression was seen to be higher in tumors and their adjacent tissues compared to the normal tissue samples. CP expression was seen to increase significantly with an increasing glioma grade in the TCGA, CGGA, Gravendeel, and Rembrandt databases. Subsequent KM curves and statistical test analyses revealed that OS was significantly lower in the High-CP category than the Low-CP in the datasets. In addition, the clinical prognostic relevance of the CP in the patients suffering from glioma was explored. A higher CP expression was significantly correlated with the glioma grade, age, chemotherapy status, IDH mutations, PRS type, and 1p19q co-deletion status in patients with glioma. COX regression analysis in the TCGA and CGGA databases suggested that CP could serve as an independent prognostic factor that affected the prognosis of the patients suffering from glioma (*p* < 0.001). Therefore, CP could be regarded as an independent prognostic biomarker for glioma and could enable the development of the targeted precision oncology.

Cellular iron is involved in several metabolic processes and is an essential microelement for cell growth and metabolism. Compared to normal cells, tumor cells required a higher iron concentration to stimulate DNA synthesis and increase cell proliferation. Disruption of iron regulatory pathways can lead to iron accumulation, thus participating in tumor mutagenesis and promoting tumor growth. For several malignancies, the degree of Fe homeostasis-linked gene expression has been used as both a prognostic indicator and a therapeutic target (Tsai et al., 2020). Some iron chelators, including DFX, desferrioxamine (DFO), Dp44mt, and triazepine, also induce apoptosis in several types of cancers and have been developed as antitumor drugs that display anticancer effects in malignancies like prostate cancer, lung cancer, neuroblastoma, leukemia, oral cancer, and breast cancer (Tan et al., 2023). In this study, genes and proteins interacting with CP were identified from the GeneMania and STRING datasets and found that iron metabolism-related genes such as SLC40A1 were closely related to CP.

TME plays a key role in the progression and invasion of gliomas. Several new treatment approaches have emerged in recent years for tumors, including immunotherapy (Hovhannisyan et al., 2023). However, the application status of immunotherapy in glioma is unsatisfactory due to disruption by the suppressive TME. During glioma progression, tumor cells evade evading host immunosurveillance and continue to grow. This process involves glioma-secreted cytokines that shape the intratumoral microenvironment and systematically alter the proliferation, differentiation, and function of immune cells in the body (Garner and de Visser, 2020). The cytokines present within the peritumoral environment are involved in the overall tumor progression process. They can not only cause immunosuppression of the tumor and evade the immune surveillance, but also promote angiogenesis and enhance the growth and invasiveness of tumor cells (Al Hrout et al., 2023). Therefore, a full understanding of the role of relevant cytokines in the TIME of glioma may provide an important theory for immunotherapy of glioma. Treatments targeting TME, like anti-PD-1 therapy and T cell immunotherapy, have achieved good outcomes. Whereas, the therapeutic effect of Immune Checkpoint inhibitors (ICBs) in glioma has been unpredictable and unfavorable, with only 8% of patients with GBM having a definite response (Arrieta et al., 2023). Currently, a growing body of data suggests that iron metabolism in TME is an important factor in maintaining cancer cell survival. Therefore, identifying iron metabolism-related factors affecting TME is crucial for the treatment of glioma.

In the present study, the relevant pathways and biological functions of CP were investigated using KEGG and GO pathway enrichment analyses to further investigate the mechanism of action of CP in glioma progression and its relationship with immune microenvironment. The results implied that CP was linked to immune function. In addition, the findings in this study revealed the correlation between the high CP expression in gliomas and increased infiltration of macrophages, B cells, neutrophils, dendritic cells, CD4^+^ T cells, and CD8^+^ T cells, with the help of the CiberSort and ESTIMATE algorithms and Timer portal. Immune and mesenchymal scores were computed for every glioma sample in CGGA to assess the proportion of immune and mesenchymal components in TME. TME scores were significantly elevated in the High-CP expression category compared to the Low-CP expression category. Based on the data derived from the TCGA database, the TMB scores were significantly elevated in the High-CP expression category compared to the Low-CP expression category. Additionally, the correlation of well-known T cell checkpoints like PD-L1, PD-1, and CTLA-4 with CP expression in the GEPIA cohort was further investigated. The findings indicated that the CP expression was related significantly to the PD-1, PD-L1, and CTLA-4 expression in LGG and GBM. In the GBM dataset of the TCIA web platform (https://tcia.at/home), patients in the high-CP expression group exhibited significant immune escape in CTLA-4 and PD-1 positive GBM tissues compared to the low-CP expression group after anti-PD-1 and CTLA-4 treatments. These findings further support the close correlation between CP expression and immune invasion, suggesting the involvement of CP in the escape of the immune cells in the glioma TME.

## 5 Conclusion

CP was confirmed to be an independent prognostic factor for predicting the prognosis of glioma patients through bioinformatics analyses of the glioma databases, and its specific mechanism may be related to tumor immunity. The present study still has many limitations. We focused on the analysis of the databases. Therefore, more *in vivo* and *in vitro* experiments need to be conducted for validating the above findings. In addition, the specific upstream and downstream pathways and mechanisms of CP need to be further explored. In conclusion, these findings suggest that CP could be regarded as a novel immune-linked therapeutic target for glioma. On the other hand, the exact role played by CP in TME deserves further investigation.

## Data Availability

The datasets presented in this study can be found in online repositories. The names of the repository/repositories and accession number(s) can be found in the article/Supplementary Material.
