# Supplementary material for: Ceruloplasmin is associated with the infiltration of immune cells and acts as a prognostic biomarker in patients suffering from glioma
Source: Front Pharmacol. 2023 Aug 11;14:1249650. doi: 10.3389/fphar.2023.1249650 (PMC10450624; doi:10.3389/fphar.2023.1249650)
Supplement: Supplementary file 1 [file DataSheet1.docx]

Supplementary Material

Ceruloplasmin is associated with the infiltration of immune cells and acts as a prognostic biomarker in patients suffering from glioma

**Miaomiao Jia, Tianyu Dong, Fanghao Rong, Jiamin Zhang, Wei Lv, Shuman Zhen, Yuming Wu, Lulu Wang*, Huixian Cui*, Peipei Hao***

*** Correspondence:** Corresponding Author: 18500795@hebmu.edu.cn

# Supplementary Figures


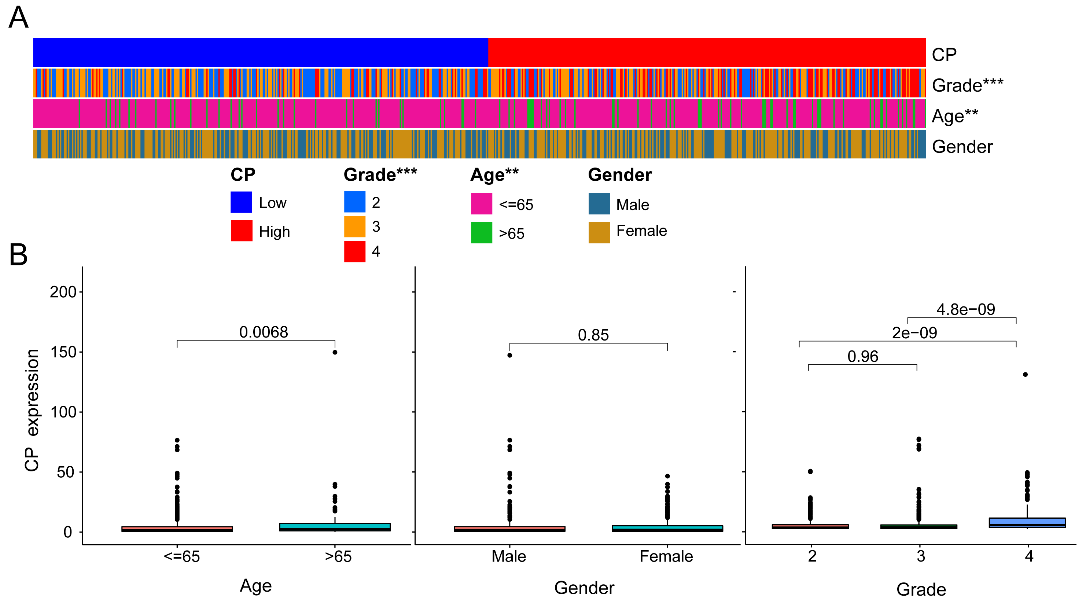


**Supplementary Figure 1.** **Analysis of the relationship between CP and clinical features of glioma samples in TCGA dataset.** (A) The relationship between CP expression and clinical features of TCGA dataset. (B) The clinical feature was analyzed for differences in the CP high/low expression groups.

**
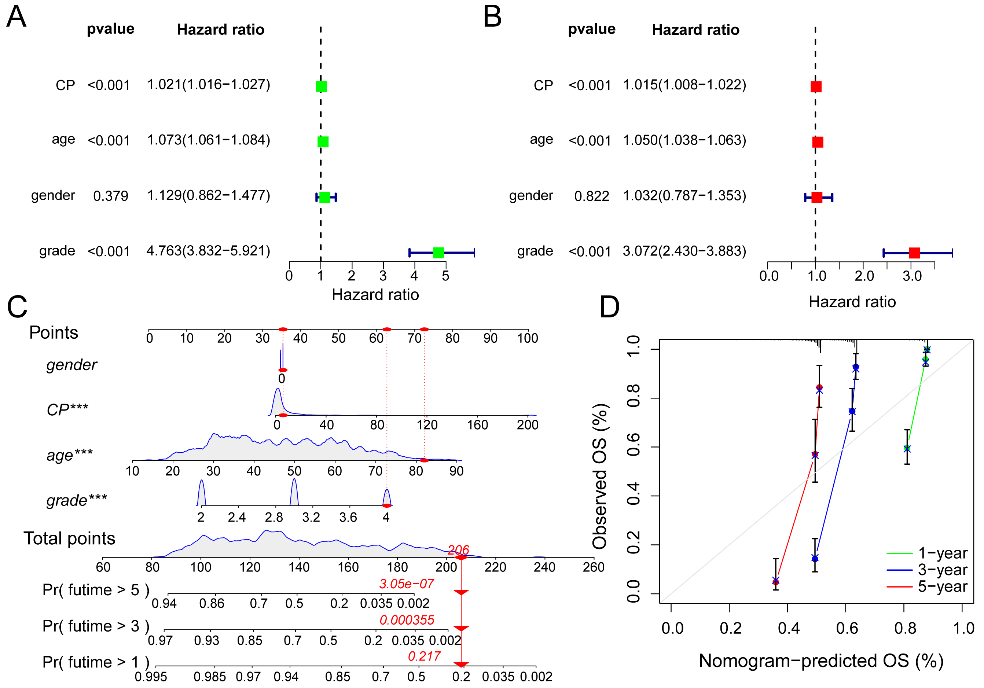
**

**Supplementary Figure 2.** **COX regression analysis and establishment of prognostic model in TCGA dataset.** (A) The univariate analysis and of CP in TCGA data set. (B) The multivariate analysis and of CP in TCGA data set. (C)The nomogram was constructed based on four factors for predicting 1 year, 3 years or 5 years survival in TCGA glioma patients. (D)The calibration plots of internal validation in TCGA showed well consistency in predicting 1 year, 3 years or 5 years survival.


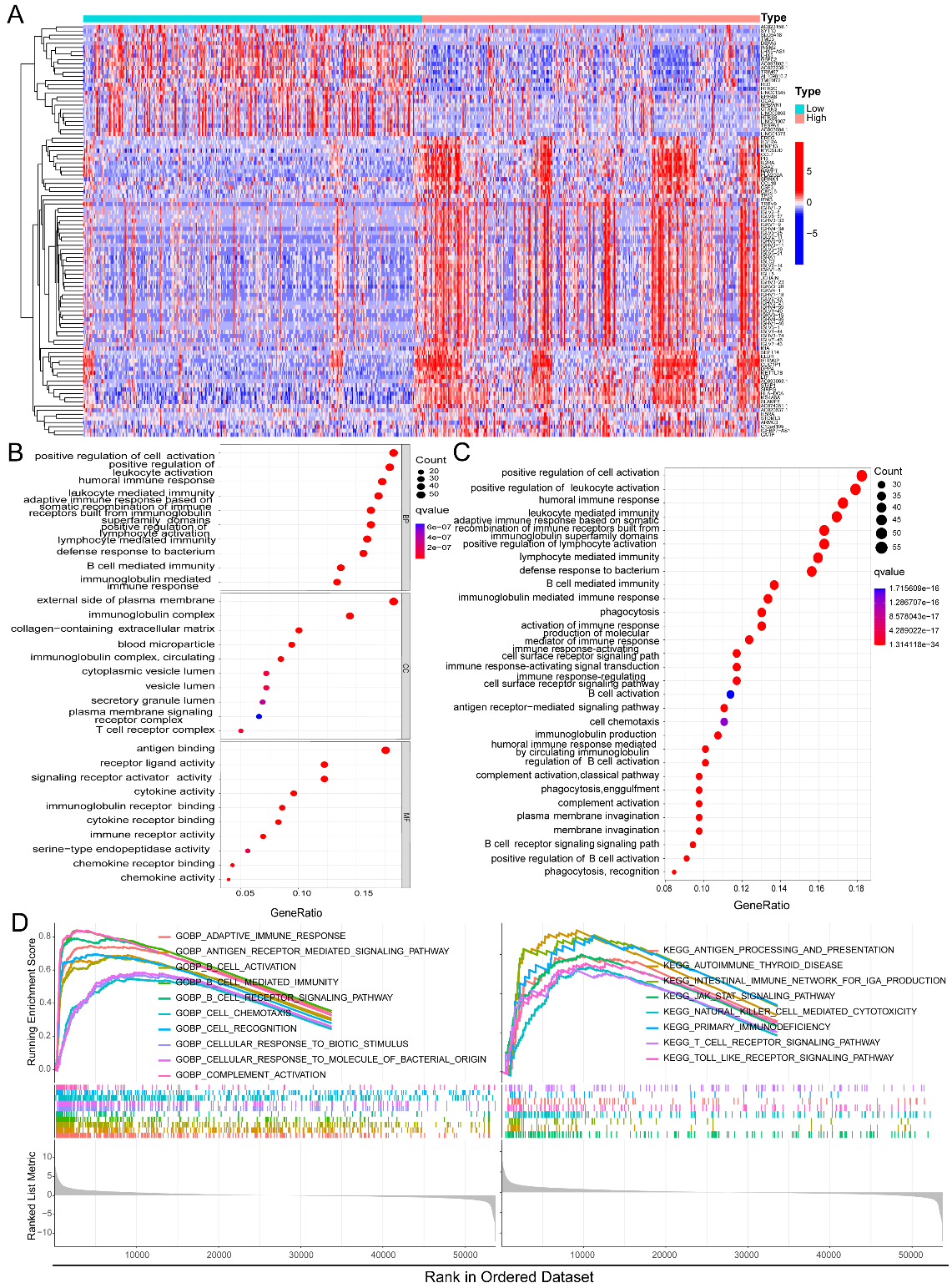


**Supplementary Figure 3 Functional analysis of DEGs in TCGA datasets between high and low CP expression groups.** (A)Heatmaps of the differential genes between high and low expression groups of CP. The GO (B) and KEGG (C) analysis of DEGs. (D)The GSEA enrichment analysis GO and KEGG of CP high and low expression groups in TCGA.
